# Knowledge, attitudes, and associated factors towards HIV pre-exposure prophylaxis among health care providers

**DOI:** 10.1038/s41598-024-56371-0

**Published:** 2024-03-14

**Authors:** Getachew Mekonnen, Tiliksew Liknaw, Alemayehu Anley, Abebe Dilie Afenigus

**Affiliations:** 1 Department of Nursing, Shebel Berenta Hospital, Shebel Berenta, Ethiopia; 2 Department of Nursing, College of Medicine and Health Sciences, Debre Markos University, Debre Markos, Ethiopia; 3 Department of Nursing, College of Medicine and Health Sciences, Arba Minch University, Arba Minch, Ethiopia

**Keywords:** Knowledge, Attitudes, Healthcare provider, And Pre-exposure prophylaxis, Diseases, Health care

## Abstract

The knowledge and attitudes of health care providers were limited as reviewed in many studies. Attitudes and knowledge about pre-exposure prophylaxis among healthcare providers have not been investigated in Ethiopia even though pre-exposure prophylaxis is a novel healthcare topic. The aim was to assess knowledge, attitudes, and associated factors towards pre-exposure prophylaxis among healthcare providers in Gojjam health facilities, North West Ethiopia, 2022. An institutional-based cross-sectional study was conducted from June 1–30 among 410 healthcare providers in public health facilities in the East Gojjam zone. A simple random sampling technique was used to recruit the required study participants. The statistical program EPI Data version 4.6 was used to enter the data, and statistical packages for Social science version 25 was used for analysis. Variables with a p-value less than 0.25 in the bivariable analysis were included in the multivariable logistic regression analysis. Statistical significance was determined with a p-value less than 0.05. The good knowledge and the favorable attitude of healthcare providers toward HIV pre-exposure prophylaxis were 55.7% (50.6–60.2%) and 60.2% (55.0–65.0%) respectively. male participant (AOR 1.67; 95% CI (1.01–2.55), service year ≥ 10 years (AOR 2.52; 95% CI (1.23–5.17), favorable attitudes (AOR 1.92; 95%CI (1.25–2.95), and providers good sexual behavior (AOR 1.85; 95%CI (1.21–2.82) were significantly associated with the good knowledge, and training (AOR 2.15; 95% CI (1.23–3.76), reading the guideline (AOR 1.66; 95% CI (1.02–2.70), and good knowledge (AOR 1.78; 95% CI (1.16–2.75) was significantly associated with the favorable attitudes. In general, the finding of this study shows that the knowledge and attitudes of healthcare providers were low. Since this is a new initiative their knowledge is lower than their attitudes. Male, service year 10 years, and good provider sexual behavior were factors significantly associated with good knowledge. Training, reading the guidelines, and good knowledge were factors significantly associated with a favorable attitudes. As a result, healthcare facilities intervention programs and strategies better target these factors to improve the knowledge and attitudes of healthcare providers. Preparing training programs to enhance knowledge and attitudes towards PrEP is recommended.

## Introduction

### Background

HIV/AIDS is among chronic infectious diseases and the primary global burden of diseases^1^. Currently, there is a new strategy to prevent newly acquired infection of the human immunodeficiency virus (HIV) for people exposed to HIV/AIDS by once-daily taking medications called pre-exposure prophylaxis. Pre-exposure prophylaxis (PrEP) is an effective prevention against HIV, approved by the World Health Organization (WHO) in 2015, and by the United States Food and Drug Administration (UFDA) in 2012^2,3^. Ethiopia approved tenofovir (TDF)/ lamivudine (3TC) HIV prevention and considered HIV PrEP as one of the six pillars for HIV prevention for HIV-negative people in 2019^4^.

PrEP has been widely regarded as a biomedical prevention strategy for highly vulnerable population groups to contracting HIV^5^. Several clinical trials have demonstrated unequivocally the effectiveness of a combination of TDF/3TC fixed-dose combination used as per the WHO recommendation in reducing the risk of HIV transmission^6^. The WHO has recently recommended its adoption as one of the strategies to combat new HIV infections among individuals at substantial risk of HIV (commercial sex workers and discordant)^7^. If taken daily by an HIV- negative individual, PrEP provides over 90% reduction in HIV acquisition^8^.

Several African countries have already approved guidelines for tenofovir plus emtricitabine, although Ethiopia approved tenofovir plus lamivudine-based PrEP for HIV prevention to individuals at substantial risk of HIV as part of combination HIV prevention despite key questions remain about how to identify and deliver PrEP to those in greatest need^4,9–11^. Throughout the continent, individuals in serodiscordant relationships and Sex workers are likely to benefit from the availability of PrEP. It has been estimated that at least three million individuals in Africa are likely to be eligible for PrEP according to WHO's criteria^12^.

Health Care professionals must be aware of and willing to administer PrEP to apply PrEP guidelines and effectively deliver PrEP to at-risk groups. Provider acceptability studies have been primarily undertaken in PrEP research studies in North America^5,8,13,14^. Understanding provider concerns and challenges to PrEP knowledge and attitudes in distinct geographical and cultural settings has the potential to increase PrEP coverage among critical populations, helping to achieve the overarching objective of regional and global HIV incidence reduction^15^.

Despite these developments, the use of PrEP among those at the highest risk of HIV acquisition has been slow. Until March 2022 only 2870 people in Ethiopia have received a prescription for PrEP out of 14,046 eligible PrEP users who attend health facilities. The estimated cumulative number of people to initiated PrEP was 16,000 to 17,000, this indicates that there is a huge gap between the eligible people and the access to PrEP^16^.

Different strategies have been implemented to cope with this problem in Ethiopia including USAIDS targets of 95–95–95, the HIV/AIDS strategic plan to end HIV transmission by 2030 and reduce HIV as a public health threat, an implementation manual for pre-exposure prophylaxis of HIV infection, also national comprehensive HIV care guideline recommends PrEP for clients at substantial risk of HIV infection^4,17^.

The Federal Ministry of Health 2019 released HIV-PrEP guidelines including oral tenofovir/ lamivudine fixed-dose as a PrEP for a person who has a risk but is not yet infected with HIV according to the clinical benefit of HIV-PrEP^18^.

Health providers that work in African countries have low knowledge of PrEP and the knowledge ranges from 3.5% in Tanzania to 67% in Kenya^5,19–25^. The magnitude of the attitudes of healthcare providers toward PrEP is not similar around the world. The pooled prevalence of providers' attitudes about PrEP is 66% in the USA, 70% in England, and 79 in Italy out of European countries^6,23,26^. Unlike European and American countries the attitudes of health providers in Africa is below 60% in Tanzania, Uganda, Botswana, and South Africa^10,24^.

HIV PrEP services should be given by a provider that has good knowledge and a favorable attitude. Despite this, if it is given by a provider with poor knowledge and unfavorable attitudes could have many consequences such as increased new HIV acquisition, poor patient adherence to ART medication, development of HIV drug resistance, decreased willingness to new PrEP users, decreased number of follow up patients, and an increase in the incidence of other sexually transmitted diseases^14,27^. It also decreases the satisfaction of substantial-risk individuals with PrEP service and decreases provider-to-user relationships^28^.

Providers who have poor knowledge and unfavorable attitudes still represent a significant barrier for substantial risk individuals seeking PrEP^29–31^. The knowledge and attitudes towards HIV pre-exposure prophylaxis among health care providers working in health care facilities in Ethiopia have not yet been studied. Therefore, this study aims to assess the knowledge, attitudes and associated factors towards HIV pre-exposure prophylaxis among healthcare providers working in East Gojjam zone health facilities.

## Methodology

### Study area, period, and design

A multi-institutional based cross-sectional study was conducted from June 1 to 30 in the East Gojjam zone, North West Ethiopia. The total number of healthcare work providers in the study area was 518^32^. Based on the Amhara Regional Health Bureau report there are eleven hospitals and 102 health centers under the zone^33^. Eleven hospitals and fifteen health centers are providing PrEP services for those substantial risk individuals^34^.

### Population

The source populations were healthcare providers (physicians, health officers, nurses, and midwives) who were working in ART, VCT, and PMTCT in the East Gojjam zone public health facilities and The study populations of this study were healthcare providers (physicians, health officers, nurses, and midwives) who were working in ART, VCT, and PMTCT during the study period.

### Inclusion and exclusion criteria

All Healthcare providers (physicians, health officers, nurses, and midwives) who were working in ART, VCT, and PMTCT during the study period were included in the study. HCPs who were sick and on annual leave at the time of data collection were excluded from the study.

### Sample size determination

The sample size was determined by using a single population proportion formula with the assumption of 59% and 67% of the overall prevalence of knowledge and attitudes of healthcare workers towards HIV PrEP from the previous studies done in Kenya respectively^25^. 95% confidence level and 5% margin of error. Based on this study the actual sample size for the study was computed by using a single population proportion formula.

$${\text{n}} = \left( {{\text{Za}}/2} \right)^{2} {\text{P}}\;\left( {1 - {\text{p}}} \right)/{\text{d}}^{2}$$

where n = sample size of the population, Z = critical value of 95% CI = 1.96, P = Proportion of knowledge or attitudes of PrEP taken as above (0.5) D = precision (marginal error) = 0.05.

The sample size of attitudes

$${\text{N}} = \left( {{1}.{96}} \right)^{{2}} \times 0.{59} \times 0.{41}/\left( {0.0{5}} \right)^{{{2} }} = {372}$$

The sample size of knowledge

$${\text{N}} = \left( {{1}.{96}} \right)^{{2}} \times 0.{67} \times 0.{33}/\left( {0.0{5}} \right)^{{2}} = {34}0$$

Then, by adding 10% for possible non-response rate the total sample size by knowledge and attitudes was 410 and 379 respectively. The sample size for the third and fourth objectives was determined using Epi-info 7 software with the assumption of 95% CI, 5% margin of error, 80% power, and exposure to an unexposed ratio of 1:1 (Table 1).

**Table 1:** Sample size calculation by third and fourth objectives.

Factors		P (%)	Power	CI	OR	Sample size	Adding 10% non-responses	Total sample size	Reference
Age (years)	≤ 34 year	81.2	80%	95%	3.7	104	11	115	^35^
	> 34 year	53.8							
Sex	Male	67	80%	95%	2.11	256	26	282	^36^
	Female	49							
Profession	Physician	70	80%	95%	2.24	228	23	251	^20^
	Nurse	51							
Service year	< 10 yrs	55	80%	95%	2.32	218	22	240	^23^
	≥ 10 yrs	74							

*CI* confidence interval, *OR* crude odd ratio, *P* proportion in percent.

Then, from the above list of sample sizes, the large sample is 410, and it was taken as the final sample of the population.

### Sampling techniques

**Figure 1:**
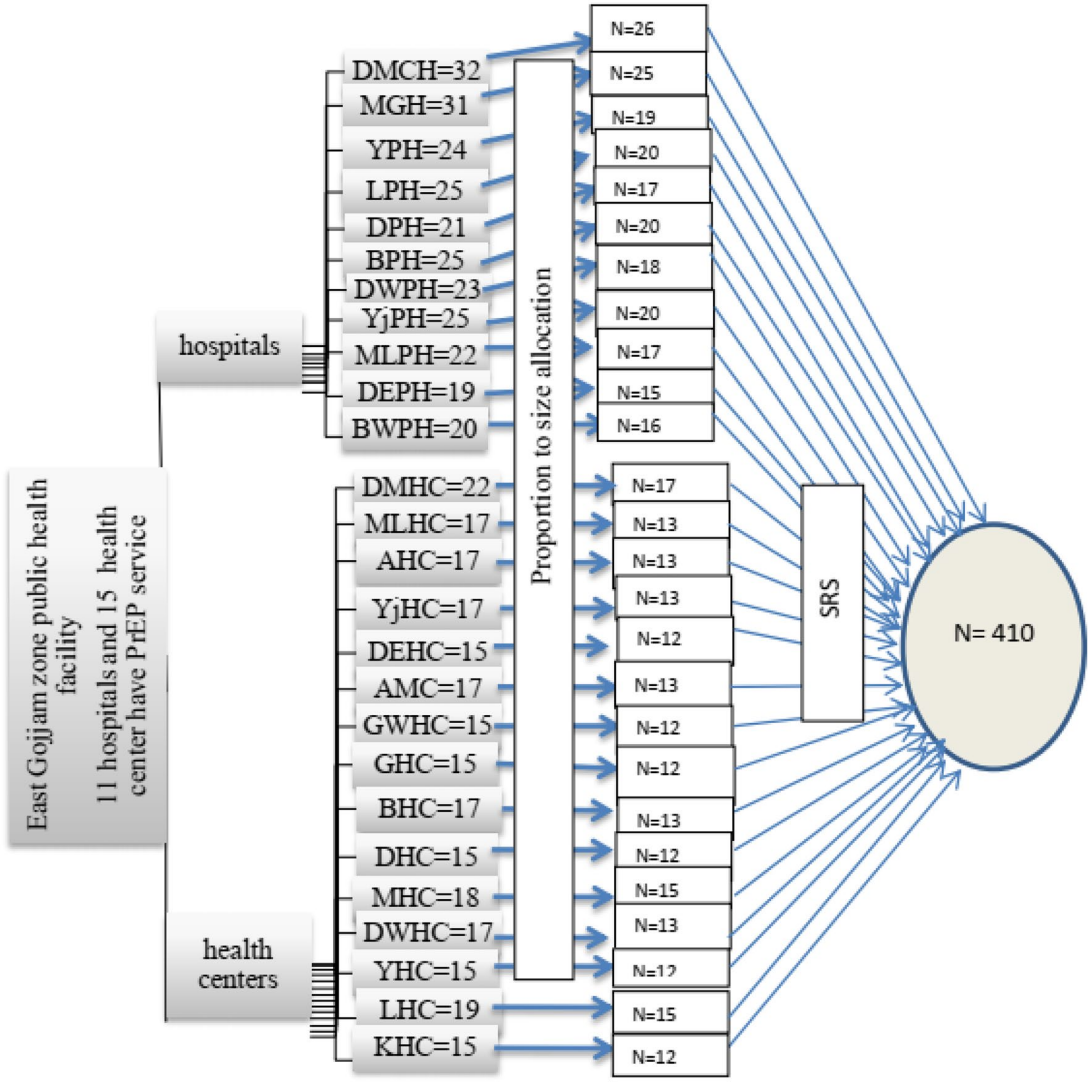
Schematic presentation of sampling procedure on knowledge, attitude, and associated factors of HIV pre-exposure prophylaxis among health providers who were working in ART, VCT, AND PMTCT in East Gojjam zone public health facility, NW Ethiopia, 2022.

A simple random sampling technique was used to select the study participants after allocating participants from public health facilities by proportional allocation to the population. The list of health providers taken from the matron and chief medical officer was used as a sampling frame and study participants were selected by lottery method until 410 health providers from a total of 518 HCPs in the working area (Fig. 1).

### Study variables

#### Dependent variables

Knowledge and attitudes of HCPs towards HIV Pre-Exposure Prophylaxis.

#### Independent variables

Socio-demographic factors: sex, age, marital status, profession, and educational level.

Sexual risky behavioral factors: Ask about sexual activity, discuss sexual behavior, offer HIV test for adults who needs treatment, refer high-risk patients, and offer PrEP for high-risk individuals.

Work-related factors: Place of work, service year, working area/ward, availability of resources, history of working in PEP, Training, and the number of patients served per day.

#### Operational definitions, data collection tools, procedures, and techniques

##### Pre-exposure prophylaxis (PrEP)

PrEP is an evidence-based HIV risk-reduction intervention with two ART drug combinations (TDF/3TC) that is offered to commercial sex workers and serodiscordant couples at risk of acquiring HIV^37^.

##### Attitudes

The attitudes of healthcare providers were measured with standardized tool and who scored greater than or equal to the mean from the total attitudes-related questions were considered to have favorable attitudes towards PrEP. There are 13 attitudes-related questions that had a Likert scale scored 1, 2,3,4, and 5 for strongly disagree, disagree, neutral, agree, and strongly agree respectively those who scored agree and strongly agree are considered to have favorable attitudes^38^.

##### Knowledge

Healthcare providers who answer ≥ 70% of total knowledge-related questions regarding PrEP have good knowledge whereas healthcare providers who scored below 70% of knowledge-related questions were considered as having poor knowledge^36^.

##### Behaviors/practices regarding providers’ HIV testing and pre-exposure prophylaxis

Someone is considered as having good Behaviors/practices regarding providers’ HIV testing and pre-exposure prophylaxis, at least if he/she answers often and always on a 5-point Likert scale from six questionnaires, and those who answered never, rarely, and sometimes were considered as having poor Behaviors/practices^39^.

Data were collected using a structured and pretested self-administered questionnaire. The questionnaire and the consent form were prepared in English. The data were collected by 11 trained BSc degree nurses and were supervised by 3 MSc nurses and 2 public health professionals having previous experience in data collection. The data were collected using a simple random sampling technique by using a lottery method and approached privately on arrival without disturbing the routine working activity in the health facility. The objective of the study was explained to them using study-specific information and enough time was given to make an independent decision to participate in the study.

### Data quality assurance and control

Data quality was controlled by giving training and appropriate supervision for data collectors. One day of training was provided to the data collectors and the supervisor on the aim of the study, how to use the questionnaire, how to approach study participants, and how to collect data and supervise.

The questionnaires were evaluated by three HIV/AIDS experts (a physician and an MSc nurse) before the data collection. The questionnaire was arranged based on the content validity index (CVI) format and sent to those experts via email to give their evaluation. Based on their responses I-CVI scores were calculated by dividing the expert agreement by the number of experts, and finally, the average of I-CVI scores across all items was computed. Based on this procedure the S-CVI was 0.86 for knowledge and 0.88 for attitudes-related questions. Which indicates the tool is acceptable.

A pretest was conducted on 5% (n = 21) of the total sample size at Finote Selam General Hospital. The reliability of the tool was checked before data collection with alpha coefficients for the knowledge, attitudes, and providers' sexual behavior scales of 0.739, 0.726, and 0.778, respectively. Continuous follow-up and supervision were also made by the principal investigator throughout the data collection period. A review was made to check the completeness of the questionnaire and corrections was made. Each questionnaire and data sheet was checked before the data entry. The data was entered daily for nearby sites and every week for those far sites and there was no identified major missing data.

### Data processing and analysis

The data were checked for discrepancies and completeness. The data were entered using Epi-data version 4.6. Then the data were cleaned, coded, and analyzed using SPSS version 25. The Multicollinearity between each independent variable was checked and there was no correlation between the independent variables with the Variance inflation factor (VIF) with a maximum value of 2.3 and the Tolerance test with a minimum value of 43.6%.

Hosmer and Lemeshow's goodness of fit test was used to test the model adequacy using a p-value (p = 0.615) for knowledge and (P = 0.269) for attitudes model fit. Variables having a p-value < 0.25 in a bi-variable analysis were entered into a multivariable binary logistic regression model to adjust for possible confounders. P-value < 0.05 and odds ratio (OR) with a 95% confidence interval were considered as a measure of statistically significant variables in this study. The result was described and expressed by using tables, graphs, and narrative descriptions.

### Ethical approval and consent to participate

Ethical clearance was obtained from the Debre Markos University, College of Health Sciences Ethical and Research review committee with an ethical clearance reference number of HSC/R/C/Ser/PG/Co/214/11/14. A formal letter of cooperation was written to eleven hospitals and other fifteen health centers. All methods were performed according to relevant guidelines and regulations. Informed consent was obtained from each study participant. Data was kept anonymously in the distributed questionnaire to keep confidentiality.

## Results

### Socio-demographic characteristics of healthcare providers

A total of 397 healthcare providers were included in the study, giving a response rate of 96.8%. Of the total participants, 314 (79.1%) healthcare providers ranged from 25 to 34 years old with a mean age of 29.48 with SD ± 4.07 years. The majority of the participants 255 (64.2%), were married and 218 (54.9%) of the participants were male by sex. Regarding the profession of participants around 213 (53.7%) were nurses, and 290 (73.0%) participants were first-degree holders (Table 2).

**Table 2:** Socio-demographic characteristics of health care providers who are working in ART, VCT, and PMTCT in East Gojjam zone public health facility, Northwest Ethiopia, 2022.

Variable	Category	Frequency	Percentage (%)
Age (years)	20–24 years	16	4.0
	25–34 years	314	79.1
	> 34 years	67	16.9
Sex	Female	179	45.1
	Male	218	54.9
Marital status	Single	128	32.2
	Married	255	64.2
	Divorced	14	3.5
Religion	Orthodox	334	84.1
	Muslim	60	15.1
	Catholic	1	.30
	Protestant	2	.50
Profession	Midwives	94	23.7
	Nurse	213	53.7
	Public health Officer	57	14.4
	Doctors	31	7.8
	Specialist	2	.50
Educational level	Diploma	85	21.4
	First degree	290	73.0
	Second degree	22	5.5

### Providers' work-related characteristics

Among 397 study participants, 205 (51.7%) participants were from the hospital, and 180 (45.3%) study participants were working in ART. 200 (50.4%) participants had work experience of 5 -10 years, but only 117 (29.5%) participants had received the training on PrEP. Two hundred sixty-eight (67.5%) of study participants read the current Ethiopian PrEP implementation guideline. The majority (97.5%) of participants heard about PrEP and the main source of information was from the health facility (Table 3).

**Table 3:** Work-related characteristics of health care providers who are working in ART, VCT, and PMTCT at East Gojjam zone public health facility, Northwest Ethiopia, 2022.

Variable	Category	Frequency	Percent (%)
Workplace	Health center	192	48.3
	Hospital	205	51.7
Working area/ward/	PMTCT	101	25.4
	VCT	116	29.2
	ART	180	45.3
been working in PEP	No	196	49.4
	Yes	201	50.6
Service year	< 5 year	142	35.8
	5–10 year	200	50.4
	> 10 year	55	13.9
Training on PrEP	No	280	70.5
	Yes	117	29.5
Have you heard about HIV PrEP	No	10	2.5
	Yes	387	97.5
Available resource	PrEP guidelines	110	27.7
	Literature	21	5.3
	PrEP medications	149	37.5
	Books	11	2.8
	All	106	26.7
Read PrEP guidelines	No	129	32.5
	Yes	268	67.5
Number of patients served per day	0–5 patients	18	4.5
	6–20 patients	257	64.7
	> 20 patients	122	30.7

**Figure 2:**
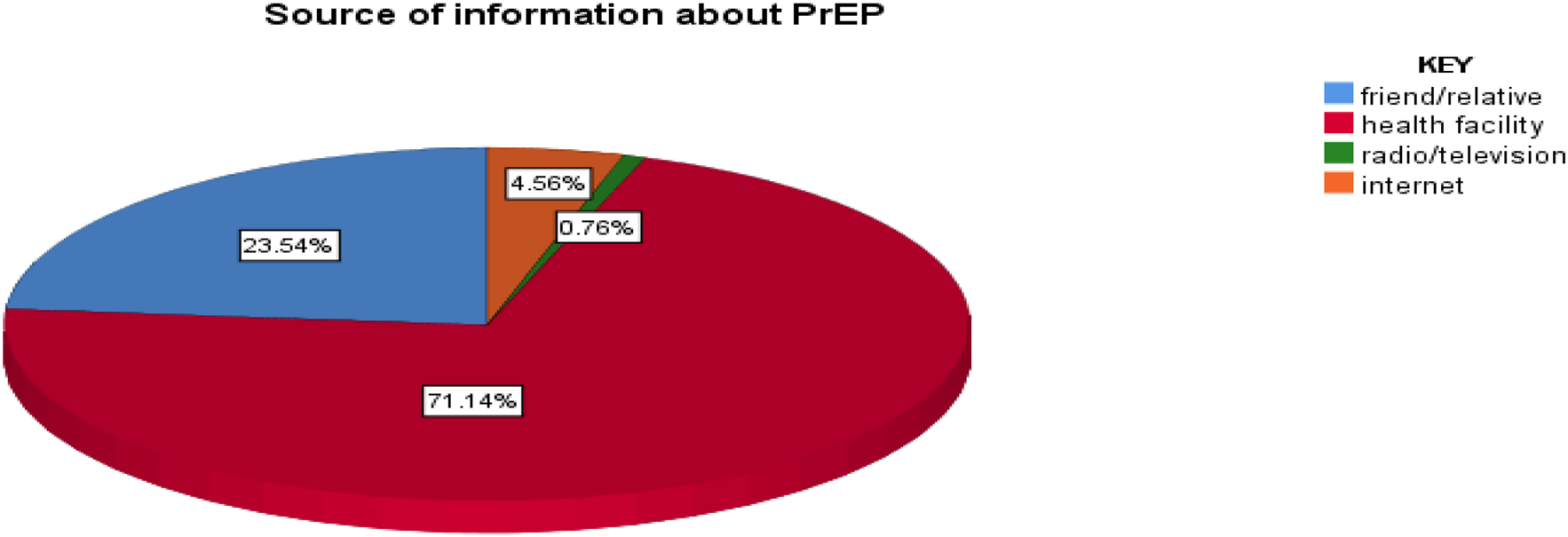
Source of information about HIV pre-exposure prophylaxis who are working in ART, VCT, AND PMTCT in East Gojjam zone public health facility HCPs, Northwest Ethiopia, 2022.

The majority of the participants get information about PrEP from a health facility (Fig. 2).

### Sexual behavior-related characteristics of healthcare workers

**Figure 3:**
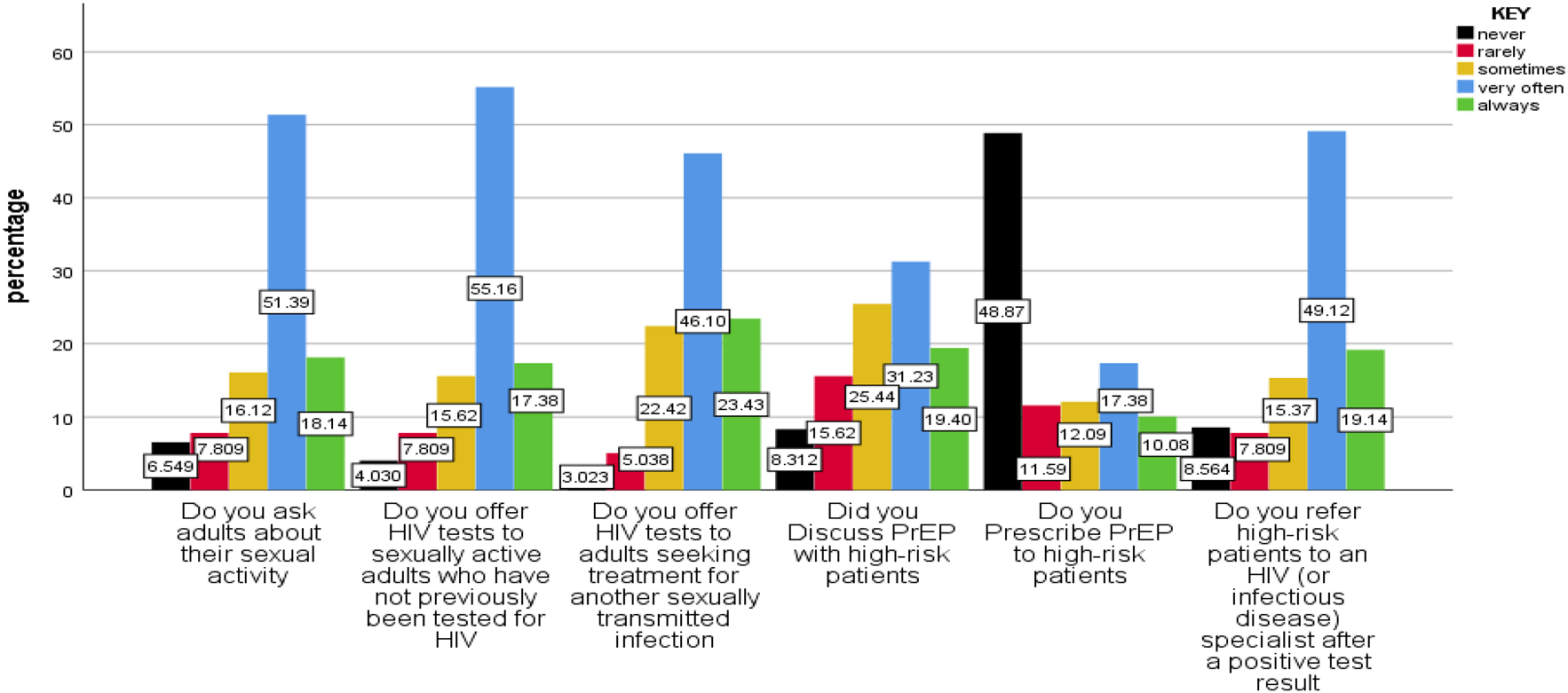
Behaviors/practices regarding providers’ HIV testing and pre-exposure prophylaxis among HCPs who are working in ART, VCT, and PMTCT in East Gojjam zone public health facility, Northwest Ethiopia, 2022.

Seventy-two (18%) and 204 (51.4%) providers reported always or very often asking adult patients about their sexual activity. Two-thirds of providers reported always or very often offering HIV tests to sexually active adults who had not been previously tested for HIV, while the remaining one-third reported sometimes, rarely, or never doing so. About 276 (69.5%) of providers were reported always or very often offering HIV tests to adults seeking treatment for another sexually transmitted infection (STI). Twelve respondents (3%) reported never offering to test such patients. Just over 267 (68%) of providers always or very often refer high-risk patients to an HIV (or infectious disease) specialist after a positive test result. Generally, study participants have good sexual behavior in linking their patients to HIV testing but they have a limitation on prescribed PrEP (Fig. 3).

### Knowledge of healthcare providers

Out of the 397 study participants, 221 (55.7%) with 95% CI (50.6–60.2) of the respondents had good knowledge, and 176 (44.3%) with 95% CI (39.8–49.4) had poor knowledge. Just below 1/3 of the providers 117 (29.47%) reported having trained in Ethiopian PrEP clinical practice guidelines, and around 221 (55.7%) of providers correctly answered 8 and above out of 11 knowledge-related questions. Of a total of 241 participants who read the guideline only 58.5% had good knowledge; also HCPs who serve greater than 20 patients per day were knowledgeable (59.4%) (Table 4).

**Table 4:** Knowledge of health care providers (working in ART, VCT, AND PMTCT) towards PrEP among healthcare providers of East Gojjam zone public health facilities, Northwest Ethiopia, 2022.

Variables	Categories	Count	Percent (%)
Aware that HIV testing is important before starting PrEP	Yes	328	82.6
	No	69	17.4
Which laboratory test is necessary for initial follow-up tests	HIV test	176	44.3
	serum creatinine	101	25.4
	widal-wiflex test	38	9.6
	screen for STI	72	18.1
	pregnancy test	10	2.5
For which of the following patients should PrEP be recommended	An HIV + client who lives lonely	56	14.1
	A serodiscordant negative couple	190	47.9
	An HIV −ve couple who has an HIV −ve partner	42	10.6
	Commercial sex workers	109	27.5
Number of PrEP drugs used for prevention	One	56	14.1
	Two	241	60.7
	Three	100	25.2
Which medication is approved for use as PrEP?	Abacavir/lamivudine (ABC/3TC)	53	13.4
	Dolutegravir/tenofovir (DTG/TDF)	104	26.2
	Tenofovir/lamivudine/doltegravir (TDF/3TC/DTG)	97	24.4
	Tenofovir/lamivudine (TDF/3TC	143	36.0
When taken daily, how efficacious is PrEP at reducing HIV acquisition risk?	50–60%	26	6.5
	61–70%	66	16.6
	71–80%	38	9.6
	81–90%	75	18.9
	Greater than 90%	192	48.4
Which of the following is an adverse effect of PrEP that requires routine laboratory monitoring?	Hepatotoxicity	142	35.8
	Reduced renal function	157	39.5
	Bone mineral density loss	49	12.3
	Dyslipidemia	27	6.8
	Anemia	22	5.5
How often do patients taking PrEP require HIV screening?	Only after potential HIV exposures	30	7.6
	Monthly	57	14.4
	Every 3 months	264	66.5
	Every 6 months	36	9.1
	Every 12 months	10	2.5
Which patients are excluded from PrEP service according to Ethiopian PrEP guideline	Patients who have HIV infection	126	31.7
	Patients with Cr level ≥ 60 ml/min	164	41.3
	Presence of HBV	54	13.6
	Patients on MDR TB treatment	53	13.4
When to stop PrEP service after initiation	After a patient HIV positive test	125	31.5
	When a patient develops a renal disease	98	24.7
	When a patient is adherent to treatment	129	32.5
	When a patient does not need PrEP	45	11.3
Recommended routine monitoring tests for all patients taking PrEP	Kidney function test	57	14.4
	Liver function test	165	41.6
	Hepatitis B serology	50	12.6
	Stool analysis	55	13.9
	HIV screen	70	17.6

Of the respondents, 82.6 percent were aware that HIV testing is important before starting PrEP and 74.75% of participants answered that commercial sex workers and serodiscordant negative couples are eligible for PrEP service. Concerning the number of drugs used for PrEP 61% of the respondents are familiar with two drugs used, but most of the health care providers lack the knowledge to differentiate which drugs and the effectiveness of the regime, 36.2% answered Tenofovir/lamivudine (TDF/3TC and (48.4%)) answered that PrEP is effectively greater than 90%. Most study participants lack knowledge related to the adverse effect, and the exclusion criteria of PrEP drugs were mostly answered below 50.0%%, which are 39.55%, 40.3%, and 48.4% respectively.

### Factors affecting the knowledge of healthcare providers

Logistic regression analyses were performed to identify factors significantly associated with good HIV-PrEP knowledge. Those variables' p values less than 0.25 (age, sex, profession, educational level, working place, working area, worked in PEP, service year, training, reading the guideline, number of patients served, attitudes, and risky sexual behavior and HIV testing practice) were entered into multivariable logistic regression. Regarding a positive knowledge of HIV-PrEP, bivariable logistic regression showed several factors were significantly associated with this parameter. Being a male, serving more than 10 years, providers having favorable attitudes and good sexual behavior activity to HIV testing were the independent factors associated with a positive knowledge of HIV-PrEP in multi-variable logistic regression analysis.

The odds of having good knowledge were 2.5 times higher among healthcare providers who have more than 10 years of experience than HCPs having less than 5 years of experience [AOR] 2.52, 95% confidence interval [CI] (1.23–5.17) P-value: 0.012. The odds of healthcare providers’ knowledge among males were 1.7 times more likely to be knowledgeable than female healthcare providers (AOR 1.67, 95% CI ( 1.01–2.55) P-value: 0.017.

The knowledge of healthcare providers was also highly associated with the attitudes of study participants, the odds of having good PrEP knowledge were nearly 2 times higher among those who had favorable attitudes as compared to healthcare providers who had unfavorable attitudes about PrEP (AOR 1.92, 95% CI (1.25–2.95) P-value:0.003.

The other significantly associated independent variable with knowledge of PrEP during multiple logistic regressions was the sexual behavior of health care providers, the odds of having good knowledge of PrEP were 1.8 times higher among providers who had good sexual behavior and HIV testing practice as compared to HCPs who had poor sexual behavior and HIV testing practice (AOR 1.85, 95% CI (1.21–2.82) P-value: 0.004 (Table 5).

**Table 5:** Factors associated with knowledge of health care providers who are working in ART, VCT, AND PMTCT in East Gojjam zone public health facility, Northwest Ethiopia, 2022.

		Knowledge		OR with 95% CI		P
		Poor knowledge	Good knowledge	COR	AOR	
Age	20–24 years	8	8	1	1	
	25–34 years	148	166	1.12 (0.41, 3.06)	0.62 (0.20, 1.93)	0.410
	> 34 years	20	47	2.35 (0.77, 7.14)	0.48 (0.12, 1.95)	0.302
Sex	Female	91	88	1	1	
	Male	85	133	1.62 (1.09, 2.42)	1.67 (1.10, 2.60)	**0.017**
Profession	Midwives	49	45	1	1	
	Nurse	91	122	1.46 (0.90, 2.38)	1.04 (.61,1.77)	0.897
	PHO	25	32	1.39 (0.719, 2.70)	0.54 (0.25, 1.17)	0.120
	Doctors	11	22	2.18 (0.95, 4.99)	1.87 (0.75, 4.70)	0.182
Educational level	Diploma	48	37	1	1	
	1st degree	123	167	1.76 (1.08, 2.87)	1.41 (.81,2.47)	.224
	2nd degree	7	15	2.78 (1.49, 4.05)	2.18 (0.62, 5.71)	0.228
Service year	< 5 year	73	69	1	1	
	5–10 year	89	111	1.32 (0.86, 2.03)	1.26 (0.80, 1.98)	0.313
	> 10 year	14	41	3.10 (1.55, 6.18)	2.52 (1.23,5.17)	**0.012**
Working area	PMTCT	53	48	1	1	
	VCT	57	59	1.14 (0.67, 1.95)	0.98 (0.20, 4.81)	0.982
	ART	66	114	1.91 (1.16,3.13)	1.18 (.24,5.79)	0.840
Working in PEP	No	102	94	1	1	
	Yes	74	127	1.86 (1.25, 2.78)	1.43 (.83,2.46)	0.196
Read PrEP guidelines	No	67	62	1	1	
	Yes	109	159	1.58 (1.03, 2.41)	0.97 (0.58, 1.62)	0.900
Training	No	139	141	1	1	
	Yes	37	80	2.13 (1.35, 3.36)	1.52 (0.89, 2.58)	0.123
Number of patients served / day	0- 5 pts	11	7	1	1	
	6–20 pts	122	135	1.74 (0.65, 4.63)	1.76 (0.63, 4.90)	0.278
	> 20 pts	43	79	2.89 (1.04, 7.99)	2.86 (0.99, 8.26)	0.052
Attitude	Unfavorable attitude	88	73	1	1	
	favorable attitude	88	148	2.02 (1.35, 3.05)	1.92 (1.25, 2.95)	**0.003**
Providers sexual behavior	Poor sexual bhr	102	79	1	1	
	Good sexual bhr	74	148	2.48 (1.65, 3.72)	1.85 (1.21, 2.82)	**0.004**

*CI* confidence interval, *COR* crude odds ratio, *AOR* adjusted odds ratio.

### Attitudes of healthcare providers toward PrEP

The mean score of the total attitudes questions was 43 with SD ± 6.58. Out of the total of 397 study participants, 239 (60.2%) with 95% CI (55.0–65.0) of the respondents had a favorable attitudes, and 158 (39.8%) with a 95% CI (34.9–44.8) had an unfavorable attitudes.

Of the respondents, 322 (81.1%) agree and strongly agree that 3TC/TDF is a safe drug to use as PrEP. One hundred forty-nine (48.6%) of participants agreed that PrEP is an effective prevention tool in the real world. In addition, 249 (62.7%) of the participants also believe that PrEP is more effective than PEP. Fifty-five percent of participants also believe that PrEP will put their patients under discrimination, 250 (63%) also agreed and strongly agreed that PrEP leads to an increase in sexually transmitted diseases (Table 6).

**Table 6:** Attitudes of health care providers who are working in ART, VCT, AND PMTCT in East Gojjam zone public health facility, Northwest Ethiopia, 2022.

Attitude questions	Strongly disagree	Disagree	Neutral	Agree	strongly agree
Believe PrEP has a greater impact than testing and care on the HIV epidemic	24	97	30	194	52
	6.00%	24.40%	7.60%	48.90%	13.10%
Believe PrEP will not affect the funding of other prevention methods	26	120	24	198	29
	6.50%	30.20%	6.00%	49.90%	7.30%
Agree with PrEP is an effective prevention tool in the real world	22	104	44	193	34
	5.50%	26.20%	11.10%	48.60%	8.60%
Believe that PrEP has little impact on ARV resistance	15	140	23	195	24
	3.80%	35.30%	5.80%	49.10%	6.00%
Believe that Patients will adhere to daily PrEP	12	127	28	197	33
	3.00%	32.00%	7.10%	49.60%	8.30%
Believe that PrEP will have a greater impact than behavioral interventions on preventing HIV infection	30	125	17	194	31
	7.60%	31.50%	4.30%	48.90%	7.80%
Believe that PrEP will lead to an increase in STIs	33	96	18	214	36
	8.30%	24.20%	4.50%	53.90%	9.10%
Do you agree with PrEP should be widely available based on current evidence	21	78	34	234	30
	5.30%	19.60%	8.60%	58.90%	7.60%
Believe that Taking PrEP will put my patients at risk of sexual or physical coercion	25	86	24	239	23
	6.30%	21.70%	6.00%	60.20%	5.80%
Believe that PrEP is more effective than PEP for frequent PEP users	11	75	26	249	36
	2.80%	18.90%	6.50%	62.70%	9.10%
Believe that Patients will not perceive to be HIV positive if taking PrEP	20	106	30	215	26
	5.00%	26.70%	7.60%	54.20%	6.50%
I am worried that my patients will be stigmatized for taking PrEP	40	153	22	165	17
	10.10%	38.50%	5.50%	41.60%	4.30%
Believe that 3TC/TDF is a safe drug to use as PrEP	13	45	17	284	38
	3.30%	11.30%	4.30%	71.50%	9.60%

### Factors affecting attitudes of health care providers toward PrEP

Logistic regression analyses were performed to identify factors significantly associated with favorable attitudes toward HIV-PrEP. There were 12 factors (age, marital status, profession, educational level, working area, worked in PEP, service year, training, reading the guideline, heard about PrEP, knowledge, and providers' risky sexual behavior) found to be significantly associated with a favorable attitude of health care providers towards HIV-PrEP in bivariable analysis, but only the following 4 factors (training, reading the guideline, good knowledge, and profession) were identified as independent factors of attitudes of health care providers towards HIV-PrEP in multivariable analysis.

The odds of having favorable attitudes among nurses were 2.7 times more favorable than medical doctors (AOR 2.70, 95% CI (1.19–6.14) P-value: 0.018. The odds of having favorable attitudes among those who take basic PrEP training had 2 times more likely favorable attitudes than healthcare providers who didn’t train in the basic PrEP guidelines. (AOR 2.15, 95% CI (1.23–3.76) P-value: 0.008.

The favorable attitudes of healthcare providers were also highly associated with reading the PrEP guideline, the odds of having favorable PrEP attitudes among those who read the guideline were nearly two times higher than healthcare providers who didn’t read about PrEP guideline (AOR 1.66, 95% CI (1.02–2.70) P-value: 0.041.

The favorable attitudes of healthcare providers were also highly associated with the good knowledge of study participants. The odds of having a favorable attitude were two times higher among those who had good knowledge as compared to healthcare providers who had unfavorable attitudes about PrEP. (AOR 1.78, 95% CI (1.16–2.75) P-value: 0.009 (Table 7).

**Table 7:** Factors associated with attitudes of health care providers who are working in ART, VCT, AND PMTCT in East Gojjam zone public health facility, Northwest Ethiopia, 2022.

Variables and category		Attitudes of HCPs		Odds ratio and confidence interval		P
		Unfavorable attitude	Favorable attitude	COR	AOR	
Age	20–24 years	9	7	1		
	25–34 years	133	181	1.75 (0.64, 4.81)	1.59 (0.54, 4.68)	0.405
	> 34 years	19	48	3.25 (1.05, 9.97)	2.20 (0.63, 7.71)	0.217
Profession	Midwives	47	47	0.94 (0.43, 2.08)	1.88 (0.75, 4.71)	0.177
	Nurse	74	139	1.77 (0.85, 3.70)	2.70 (1.19, 6.14)	**0.018**
	PHO	24	33	1.29 (0.55, 3.06)	1.28 (.51, 3.22)	0.599
	Doctors	16	17	1		
Educational level	Diploma	37	48	1		
	1^st^ degree	120	170	1.09 (0.67, 1.87)	1.03 (0.61, 1.76)	0.909
	2^nd^degree	8	14	1.35 (1.08, 6.21)	1.28 (0.96, 3.76)	0.056
Working area	PMTCT	53	48	1		
	VCT	51	65	1.41 (0.82, 2.41)	0.85 (0.19, 3.90)	0.835
	ART	57	123	2.38 (1.44, 3.93)	1.01 (0.22, 4.69)	0.992
worked on PEP	No	90	106	1		
	Yes	71	130	1.56 (1.04, 2.33)	0.88 (0.54, 1.41)	0.583
Service year	< 5 year	61	81	1		
	5–10 year	82	118	1.08 (0.70, 1.67)	0.76 (0.46, 1.27)	0.298
	> 10 year	18	37	1.55 (0.80, 2.98)	0.91 (0.36, 2.33)	0.851
Training	No	131	149	1.00		
	Yes	30	87	2.55 (1.58, 4.11)	2.15 (1.23, 3.76)	**0.008**
Read the guideline	No	74	70	1		
	Yes	87	166	2.01 (1.33, 3.06)	1.66 (1.02, 2.71)	**0.041**
Knowledge	Poor knowledge	88	88	1		
	Good knowledge	73	148	2.03 (1.35, 3.05)	1.78 (1.16, 2.75)	**0.009**
providers sexual behavior	Good sexual behavior	97	84	1		
	Good sexual behavior	64	152	2.74 (1.82, 4.15)	1.46 (0.93, 2.31)	0.103

## Discussion

In this study, the majority of participants 369 (92.9%) with 95% CI (90–95.3) heard about PrEP for HIV which is higher as compared to previous studies like research conducted in Deland Florida (75%), and in Rwanda, 86.4% of respondents heard about HIV PrEP^39,40^. This might be due to the difference in the period of adoption of the program and training opportunities.

In this study, the good knowledge of health care providers about HIV PrEP was 55.7% (50.6–60.2%), which was similar to the study conducted in America among Air Force health care providers (55%)^36^, Australia (51%) and in International AIDS Society-USA (IAS-USA) (51%)^28^. However, it is lower than the study done in the United Kingdom (80%)^20^, Tennessee Medical Center in America (78.75%)^8^, Italy (64.5%)^23^, and study in Florida 79%^39^. The possible explanation for this difference could be the tool (self-rated questionnaire) they used to rate the knowledge, countries that haven’t specific policies related to PrEP, and the other explanation could be study participants in the UK and America had an experience of being included in the previous study^8^.

On the other hand, health care providers who had good knowledge (55.7%) are higher than the study conducted in Boston Medical School (44%)^26^ and Rwanda < 50%^40^. This variation might be due to the study period, data collection method (they use a convenient sampling technique), late introduction of PrEP service, and then conducted in a small (51) study participants.

This study also found that healthcare providers who had work experience greater than or equal to 10 years had a significant association with a good knowledge of PrEP. HCPs who had this much experience were 2.5 times more likely to be knowledgeable than those who had less than 10 years of work experience (AOR 2.52). This study result is similar to the study conducted in New England^26^ and the USA Air Force^36^. The possible explanation could be health care providers who have more experience have a chance to participate in training and workshops related to HIV prevention strategies like PrEP, so greater engagement with certain continuing education tools and training influences PrEP knowledge. The other explanation might be young sexually active patients interested in PrEP may be more likely to seek out highly experienced HCPs^41,42^.

The other finding of this study was the significant association between the knowledge of PrEP and the sex of participants (males were approximately two times more likely knowledgeable than female healthcare providers (AOR 1.67). This study is in line with the study conducted in the UK and USA, which stated that there was a statistically significant difference in the proportion of male and female respondents who rated their knowledge of PrEP as high or medium.^20,35,36^.

The knowledge of healthcare providers was also highly associated with the attitudes of study participants. Those providers who had favorable attitudes to PrEP had a great association with a good knowledge of PrEP (AOR 1.92). This result is in line with the study conducted in Mexico and Brazil^43^ as well as in Florida^39^. The reason could be those healthcare providers who have a favorable attitude are eager to welcome the program and read the studies related to PrEP. The other reason might be providers who have favorable attitudes may read literature related to the efficacy and side effects of PrEP medication to increase the adherence of their patients^44^.

HCPs who had good sexual behavior were 2 times more likely to have a favorable attitude than those who had poor sexual behavior (AOR 1.85). This study is in line with a study conducted in Thailand and America^38,45^. The study found that discussing sexual risk history with patients, frequency of taking a sexual history, ever prescribing antiretroviral for HIV prevention, and offering patients HIV testing and linking to STI clinics for high-risk patients were significantly associated with independent variables with knowledge of PrEP^46^. The possible explanation could be those providers read different literature about HIV prevention strategies to cascade their daily activities or to answer questions raised by their patients^21^. The other reason could be those practicing sexual behavior activities had training on HIV prevention methods.

The other finding in this study was that 49% (45.6–55.7%) of healthcare providers discussed PrEP with high-risk patients, which is in line with the study in Florida (50%)^39^, greater than the study conducted in the US navy (29.9%)^46^. But it is lower than the study conducted in Italy (69%)^23^. This difference might be providers’ perception that patients do have high-risk behaviors and most patients have a low risk for infection and it may include a lack of training regarding PrEP, such as how to offer PrEP^47^**.** Another possible explanation is response bias, for example, providers know that they should be discussing and prescribing PrEP to high-risk patients according to practice guidelines, so they indicated that they were willing to do so when additional factors impact compliance in practice^23^.

The other pertinent finding of this study was the attitudes of health care providers about HIV PrEP was 60.2% (55.0–65.0%), which was similar to the study conducted in Tanzania (61%)^24^, and the multicenter study in Uganda, Botswana, and South Africa, which is less than 60%^10^. However, this finding is less than the study conducted in America (pooled prevalence of provider attitudes was 68%)^48^ and Italy (79%)^23^ The possible explanation for this difference could be the tool (self-rated questionnaire) they used to rate the attitudes. The other explanation could be the time of adoption of the program, those countries have accepted the PrEP program since 2015^9^, so providers had a chance to get training and symposiums related to HIV prevention. However, the current study was conducted after 3 years of implementation in Ethiopia.

Similarly, these studies are also lower than the study done in Belgium (78.4%)^49^, South Africa (79%), and sub-Saharan African countries (66%)^10,44^. The possible explanation for this difference was that to study participants this study believed that promoting HIV testing and treating HIV-infected patients is more important than offering PrEP. On the other finding, this result was much greater than the study conducted in Rwanda, which is 40%. This discrepancy related to study participants in that study believed that PrEP leads patients to highly risky sexual behavior (59.3%) and is related to an increase in sexually transmitted diseases (58.3%)^40^.

The finding of this study showed that significant association between the attitudes of HCPs and the formal training of PrEP (trained HCPs were 2 times more likely favorable attitudes than untrained health care providers (AOR 2.15). This study is in line with the study conducted in Kenya^42^, Tanzania^24^, Nigeria^41^, and South Africa^44^. The possible explanation for this association is that formal training is the key to recording accurate and reliable data (efficacy; adherence, the side effect of PrEP) and it increases the attitudes of HCPs^28^.

The favorable attitudes of health care providers were also highly associated with reading the PrEP guideline, Study participants who read the guideline were approximately two times more likely to have a favorable attitude about HIV pre-exposure prophylaxis as compared to health care providers who didn’t read about PrEP guideline (AOR 1.66). This finding is in line with the study conducted at Tennessee Medical Center^8^, Florida^39^, Germany^22^, and Kenya^25^. The possible reason might be those who read the guideline get a clear picture of PrEP and more detailed information about it, so HCPs who read the guideline might have a favorable attitude^9^.

The main concerns about PrEP found in our study were adherence to PrEP and long-term side effects, and it increases risky sexual behavior, which has also been found in other studies in Belgium. As found in other studies, addressing the belief that PrEP is an effective intervention to prevent HIV^4,37^ and that it is a good prevention strategy may be crucial to enhancing the acceptance of PrEP among HCPs.

The favorable attitudes of healthcare providers were also highly associated with the knowledge of PrEP, knowledgeable participants had a favorable attitude about HIV pre-exposure prophylaxis as compared to healthcare providers who did not know about PrEP (AOR 1.78). This finding is in line with the study conducted at Tennessee Medical Center^8^, Germany^22^, and Kenya^25^. This finding may be explained by that less knowledgeable physicians possibly had more HIV-PrEP concerning issues about medication adherence behavior and risk of developing HIV drug resistance in real-life clinical practice^38^.

A finding that was associated with a favorable attitude towards PrEP was profession (nurses were more likely to have a favorable attitude by three times as compared to medical doctors (AOR 2.7). This study is in line with the study conducted in Washington^20^, and Thailand^36^, which found that nurses (73%) have a higher attitude toward HIV PrEP than physicians. This discrepancy may be due to the preview paradox (physicians believing that PrEP services must be given by other HCPs like nurses and clinical officers) and this paradox leads them to an unfavorable attitude^15^.

## Conclusion and recommendations

In general, the finding of this study shows that the knowledge and attitudes of healthcare providers who were working in East Gojjam zone facilities were low, even more than half of healthcare providers in the East Gojjam zone had good knowledge and a favorable attitude. This study showed that sex (men), service years greater than 10 years, attitudes, and sexual behavior were found to be significantly associated with the PrEP knowledge of healthcare providers. Profession (being a nurse), training, reading the guidelines, and good sexual behavior were significantly associated with the favorable attitudes of healthcare providers towards HIV pre-exposure prophylaxis.

Highly experienced HCPs should share their experience with those who have less experience. Training to female HCPs and clinicians with limited work experience should undergo PrEP-specific training to increase and promote the use of PrEP among high-risk groups and reduce the risk of HIV infections. Health facilities should make available PrEP guidelines within their workplace and standardize written PrEP. The study focuses on the knowledge and attitudes of HCPs in geographically limited areas so, we recommend that future researchers study nationwide by including the practice.

### Limitations of the study

The study may be subjected to the response set bias from the respondents. In addition to the above limitation, the sexual behavior component may have been influenced by social desirability bias.

## Data Availability

The datasets used and/or analyzed during the current study are available from the corresponding author upon reasonable request.

## Abbreviations

AGYW: Adolescent girls and young women
AIDS: Acquired immune compromised syndrome
CVI: Content validity index
FMOH: Federal Ministry of Health
HCPS: Health care providers
I-CVI: Item content validity index
PLWA: People living with AIDS
PrEP: Pre Exposure prophylaxis
SHAs: Sexual health advisors
TDF/3TC: Tenofovir Disoproxil Fumarate + Lamuvidine
UNAIDS: United Nations Program on HIV/AIDS
